# Change in hippocampal theta activity with transfer from simple discrimination tasks to a simultaneous feature-negative task

**DOI:** 10.3389/fnbeh.2014.00159

**Published:** 2014-06-03

**Authors:** Yuya Sakimoto, Shogo Sakata

**Affiliations:** ^1^Department of Systems Neuroscience, Graduate School of Medicine, Yamaguchi UniversityYamaguchi, Japan; ^2^Department of Behavioral Sciences, Graduate School of Integrated Arts and Sciences, Hiroshima UniversityHiroshima, Japan

**Keywords:** hippocampal theta activity, simultaneous feature-negative task, simple discrimination task, rat, hippocampal-independent strategy

## Abstract

It was showed that solving a simple discrimination task (A+, B−) and a simultaneous feature-negative (FN) task (A+, AB−) used the hippocampal-independent strategy. Recently, we showed that the number of sessions required for a rat to completely learn a task differed between the FN and simple discrimination tasks, and there was a difference in hippocampal theta activity between these tasks. These results suggested that solving the FN task relied on a different strategy than the simple discrimination task. In this study, we provided supportive evidence that solving the FN and simple discrimination tasks involved different strategies by examining changes in performance and hippocampal theta activity in the FN task after transfer from the simple discrimination task (A+, B− → A+, AB−). The results of this study showed that performance on the FN task was impaired and there was a difference in hippocampal theta activity between the simple discrimination task and FN task. Thus, we concluded that solving the FN task uses a different strategy than the simple discrimination task.

## Introduction

The conflict resolution model, proposed by Gray and McNaughton (2000), suggested that the hippocampus was important for providing the inhibition of a response to a stimulus that presents a conflict of goals; specifically, it plays a role in increasing the weight of negative information. Recently, Chan et al. (2001), and Davidson and Jarrard (2004) expanded on this theory by proposing that in associative learning, inhibition of a response to a conflicting stimulus occurred when a stimulus comprises simple inhibitory associations between events embedded in concurrent simple excitatory associations. They asserted that solving a negative patterning task used a hippocampal-dependent strategy, whereas solving a simple discrimination task and simultaneous feature-negative (FN) task used a hippocampal-independent strategy. During a negative patterning task (A+, B+, AB−; Sakimoto and Sakata, 2013; Sakimoto et al., 2013a,b), a rat's lever-press response for a single stimulus (A or B) was rewarded during the reinforcement trial (RFT), but a rat's lever-press response for a compound stimulus (AB) was not rewarded during the non-reinforcement trial (non-RFT). In this task, although stimulus A or B presented alone signaled a lever press response, these stimuli presented simultaneously signaled to inhibit the go-response. These stimuli had a conflicting feature. In a simple discrimination task (A+, B−), animals were rewarded when presented with a stimulus (A+), but not when a stimulus B was presented (B−). These stimuli, A or B, did not have conflicting features. Moreover, in the FN task (A+, AB−), animals were rewarded when presented with a stimulus (A+), but not when a compound stimulus AB was presented (AB−). Several researchers (Rudy and Sutherland, 1995; Chan et al., 2001; O'Reilly and Rudy, 2001; Davidson and Jarrard, 2004) supposed that the FN task used a hippocampus-independent strategy because it could be accomplished in a similar way as the simple discrimination task (specifically, that both tasks depended on the discrimination between stimulus A and B). Further, some studies showed that the loss of hippocampal function did not impair performance on the FN task (Solomon, 1977; Chan et al., 2003).

Recently, we compared hippocampal theta activity between the negative patterning task and simple discrimination task (Sakimoto and Sakata, 2013; Sakimoto et al., 2013a,b), and revealed that hippocampal theta activity transiently declined during the response inhibition of a compound stimulus in the negative patterning task, but not the simple discrimination task (Sakimoto and Sakata, 2013; Sakimoto et al., 2013b). We first discovered the relationship between hippocampal theta activity and response inhibition for the conflicting stimulus. However, we also observed a reduction in hippocampal theta activity during the response inhibition of a compound stimulus presented during the FN task (Sakimoto and Sakata, 2013). Furthermore, we revealed that the number of sessions required for a rat to completely learn a task differed between the FN and simple discrimination tasks (Sakimoto and Sakata, 2013; Sakimoto et al., 2013a). These results suggest the possibility that animals use a different strategy for solving the FN and simple discrimination tasks. In this study, we provide supportive evidence that solving the FN and simple discrimination tasks involve different strategies by examining changes in hippocampal theta activity and performance in the FN task after transfer from the simple discrimination task (A+, B− → A+, AB−). We hypothesized that hippocampal theta activity and performance on the FN task would not be affected when transferring from the simple discrimination task, if solving the FN task uses the same strategy as the simple discrimination task.

## Materials and methods

### Subjects

Twelve 3-month-old, naïve, male Wistar albino rats were included in this study. All rats were housed in individual cages and kept on a 12:12-h light-dark cycle (lights on at 8:00 am). Throughout the experiment, all rats were maintained at 85% of their *ad-libitum* weights and water was freely available. All procedures for animal treatment and surgery were conducted in accordance with the regulatory standards of Hiroshima University.

### Apparatus

This information has been described in our previous study (Sakimoto and Sakata, 2013; Sakimoto et al., 2013c). Behavioral training and electroencephalogram (EEG) recording sessions were conducted in a standard operant chamber (ENV-007 CT; MED Associates, Inc., USA). The chamber was housed in a soundproof, electrically shielded room. For delivery of 45-mg food pellets, a cup was located in the center of the front wall at floor level and a light bulb (ENV-215; MED Associates, Inc.) was mounted over the cup to provide constant illumination. A lever was positioned on the left side of the front wall. A white super luminosity LED light (41 lux) was mounted on the ceiling to present the light stimulus. A tone (2000 Hz, 75 dB) was provided via a speaker placed on the interior shell. All events were controlled, and behavioral data was recorded on a personal computer (Epson MT7500).

### Procedure

Rats were habituated to the operant box for 30 min, and were then trained to press a lever. We used 45-mg food pellet (Bio-Serv, product #F0165) as reinforcement. Following the acquisition of this response, rats were given 2 days of continuous reinforcement training (100 reinforcements/day), followed by 3 days of training at variable intervals of 20 s (VI20; 40 reinforcements/day). Next, rats were trained on the simple discrimination task. Following task training, electrodes for EEG recording were implanted into each rat, and following a 1-week recovery period, the retention test for the simple discrimination task and the FN task were conducted. EEG readings were recorded throughout the retention test of the simple discrimination task and then for the FN task.

### Learning tasks

#### Simple discrimination task

The protocol of the simple discrimination task has been used and described in our previous study (Sakimoto and Sakata, 2013). In the simple discrimination task, rats were trained to discriminate between two individually presented stimuli (tone or light). Rats were randomly assigned to one of two groups. For one group, rats' lever-press responses were rewarded when the tone stimulus (T: 2000 Hz, 75 dB) was presented, but not when the light stimulus (L: five white LED) was presented (T+, L−). For the other group, the stimuli were reversed (L+, T−). Each session consisted of 120 trials made up of 60 reinforcement trials (RFTs) and 60 non-reinforcement trials (non-RFTs). All stimuli remained on until either 10 s had elapsed or until the rat pressed the lever. Each trial was separated by variable inter-trial intervals (20–40 s). The stimuli sequences were randomly determined, but no more than four trials of the same type occurred in succession. We calculated the response rates for RFT (number of lever presses for RFTs in a session/number of total RFTs in a session) and non-RFT (number of lever presses for non-RFTs in a session/number of total non-RFTs in a session). The task criteria were met when the RFT response rate reached at least 90%, and the non-RFT response rate was no more than 50%. Learning was considered complete when the criteria were met for three consecutive days or for a total of 5 days. After reaching these criteria, all rats were implanted with electrodes for EEG recording. Following a 1-week recovery period, the retention test for the simple discrimination task was conducted for 3 sessions.

#### Simultaneous feature-negative (FN) task

After the retention test for the simple discrimination task, the rats were trained on the FN task. The FN protocol was used and described in our previous study (Sakimoto and Sakata, 2013). The rats were assigned to one of the two types of FN discriminations. For one group (T+, L−), rats were trained in the lever responses for tone stimuli (T+) but not for compound stimuli that simultaneously presented tone and light (TL−). For the other group (L+, T−), rats were trained lever responses for light stimuli (L+) but not for compound stimuli (TL−). All stimuli remained on until either 10 s had elapsed or until the rat pressed the lever. Each trial was separated by variable inter-trial intervals (20–40 s). The stimuli sequences were randomly determined, but no more than four trials of the same type occurred in succession. We calculated the response rates for RFT (number of lever presses for RFTs in a session/number of total RFTs in a session) and non-RFT (number of lever presses for non-RFTs in a session/number of total non-RFTs in a session). The task criteria were met when the RFT response rate reached at least 90%, and the non-RFT response rate was no more than 50%. Learning was considered complete when the criteria were met for three consecutive days or for a total of 5 days. In this experiment, task acquisition was divided into two stages: the early learning stage, comprising the first three sessions of discrimination training, and the late learning stage, comprising the last three sessions when the criteria of this task were met completely.

### Electrode implantation

After they were deeply anesthetized with thiamylal sodium (50 mg/kg, i. p.), the rats were placed in a stereotaxic apparatus (Narishige, Japan). EEG was recorded using the bipolar method, with the recording electrodes implanted stereotaxically in the hippocampal region, 2.4 mm below the skull surface, 3.5 mm posterior to bregma, and ±2.0 mm lateral to the midline. The reference electrodes were attached to the skull 6.0 mm anterior to bregma and +2.0 mm lateral to the midline. Polyurethane-insulated stainless steel wire electrodes (200-μm diameter; Unique Medical Co., LTD., Japan) were used as the recording electrodes, and polyurethane-insulated silver-ball electrodes (1-mm diameter; Unique Medical Co., LTD., Japan) were used as the reference electrodes. The electrodes were terminated by pin-type connectors, which were connected to sockets attached to the skull over the hippocampus using anchor screws and dental cement.

### Analysis

#### Electroencephalogram recording and analysis

**Figure 1:**
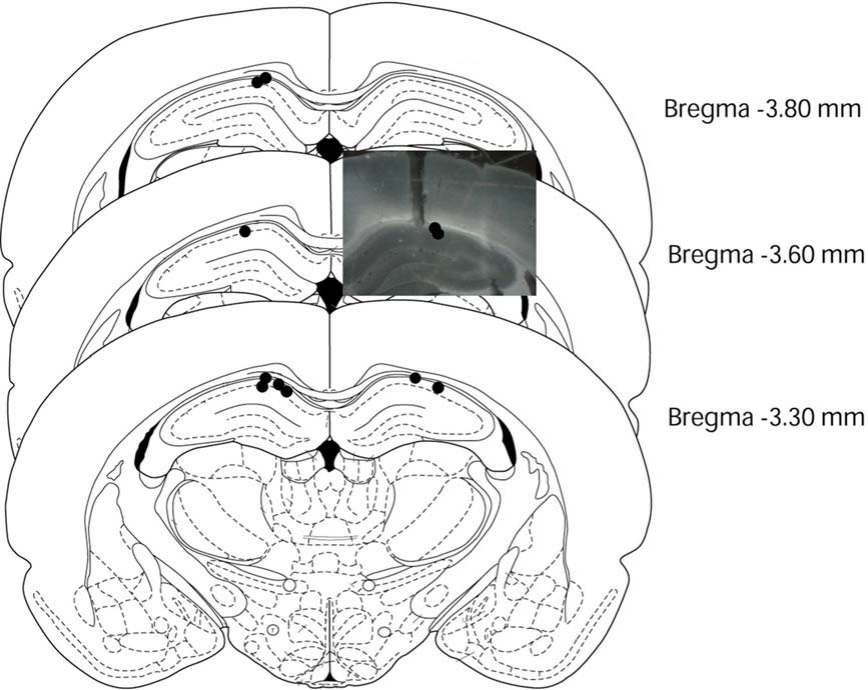
**Electrode placements in the rat brains**. This figure was modified from Paxinos and Watson (1997). Black circles and photo data indicated the placement of the electrode tips in each rat (*n* = 12).

Due to the long-term nature of the study, although a few rats (*n* = 3) were identified by bilateral recordings, but almost rats (*n* = 9) were identified by only unilateral recording at the end of the experiment. After the experiment, we used slice microscopy to determine the electrode position. In histology, all nine rats that were recorded unilaterally indicated an electrode implanted in the correct position and all three rats that were recorded bilaterally showed only one of the two electrodes implanted in the correct position. Hence, we only analyzed unilateral hippocampal EEGs from all 12 rats (Figure 1). EEG waveforms were amplified (System 360; NEC Sanei, Tokyo, Japan) and digitized at a sampling rate of 1000 Hz using a time constant of 3 s. All EEG analyses were carried out with MATLAB version R2007b (The MathWorks Inc., Natick, MA, USA), and focused on the retention test, three sessions of the simple discrimination task, and the early learning and late stages of the FN task. The recording period ran from 4000 ms before stimulus onset to 10000 ms after stimulus onset, while the analysis period spanned from 500 ms before stimulus onset to 4000 ms after stimulus onset. Hippocampal theta power was computed by wavelet analysis using a 2-ms point size. Wavelet analysis employed the Morlet basis function to determine the power of theta oscillatory activity (Sakimoto and Sakata, 2013). The analysis period was divided into 45 sub-periods of 100 ms each. Each epoch contained wavelet analysis data from 50 points; mean hippocampal theta power was computed for each period. The mean hippocampal theta power from 500 to 400 ms (−400-ms period) before stimulus onset was counted as the baseline (no stimuli were present during this period), and the relative theta power calculated for each period was normalized to that during the −400-ms period (relative theta power of each period = theta power of each period/theta power at the −400-ms period). We then analyzed the 6- to 12-Hz frequency band of hippocampal theta waves. The analysis of hippocampal EEGs included counting the number of correct responses for both RFTs and non-RFTs. A lever-press response trial for RFT and lever-press response inhibition trial for non-RFT were defined as the correct response. Trials with artifacts were eliminated from wavelet analyses.

#### Statistical analysis

Behavioral data were analyzed to examine the difference in response rate and reaction time in RFTs and non-RFTs, and compared to the retention test for the simple discrimination task and the early learning and late stages of the FN task. The response rate was assessed using an ANOVA with trial type (RFT and non-RFT) and learning stages (retention test for the simple discrimination task, early learning and late learning stages of the FN task) as within-subjects factors. The change in hippocampal theta activity over time (from −400 to 4000 ms, divided into 45 epochs of 100 ms each) was compared between the three stages (retention test of the simple discrimination task, early learning, and late learning stages of the FN task) as a within-subjects factor on each trial type: RFT and non-RFT. Multiple comparisons were corrected with the Bonferroni's method (α = 0.05).

## Results

### Behavioral data

The mean number of sessions required to achieve complete learning of the simple discrimination task was 6.25 ± 1.60 (mean ± *SD*) sessions; 8.67 ± 4.62 sessions were required to achieve complete learning of the FN task after the retention test for the simple discrimination task. Only one rat was excluded from the analysis of behavior and hippocampal theta activity in the early learning stage of the FN task because it did not require multiple sessions to learn the FN task. Another rat required two sessions to complete learning the FN task; therefore, we analyzed behavioral data and hippocampal theta activity of two sessions in the early learning stage of the FN task. All other rats (*n* = 10) required at least three sessions to completely learn the FN task.

**Figure 2:**
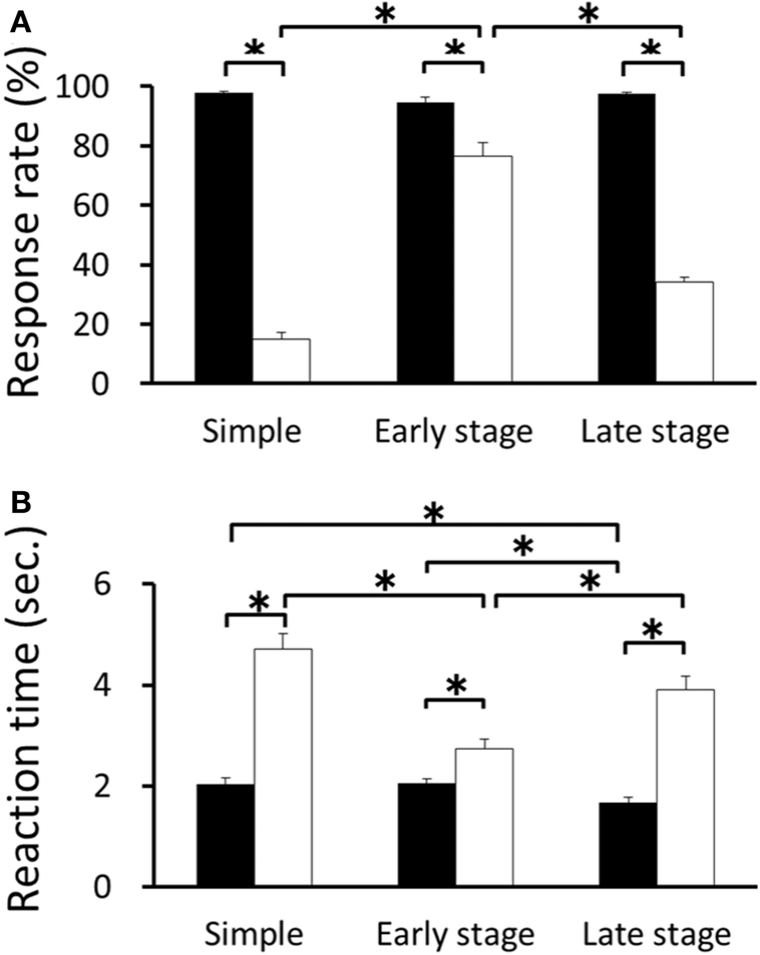
The mean response rate (A) and reaction time (B) for RFT and non-RFT during recording sessions for the retention test for the simple discrimination task (Simple), early learning stage (Early stage), and late learning stage (Late stage) of the FN task (^*^p < 0.05). Error bars indicate s.e.m.

The mean response rates for RFT and non-RFT were 97.98 ± 1.52% and 14.19 ± 7.13% in the retention test for the simple discrimination task, 94.70 ± 5.54% and 76.62 ± 14.45% in the early learning stage of the FN task, and 97.37 ± 2.02% and 33.89 ± 6.09% in the late learning stage of the FN task, respectively. The Two-Way repeated measures ANOVA revealed a significant trial type (RFT and non-RFT) × stage (retention test of the simple discrimination task, early learning stage of the FN task, late learning stage of the FN task) interaction [*F*_(2, 20)_ = 139.06, *p* < 0.001; Huynh-Feldt ε = 0.70]. *Post-hoc* tests revealed that the response rate in the RFTs was significantly higher than that in the non-RFTs in the three stages (all *p* < 0.05). Moreover, the response rate in the non-RFTs in the early learning stage of the FN task was significantly higher than that in the retention test for the simple discrimination or late learning stages of the FN task (all *p* < 0.05; Figure 2A). The mean reaction time for RFT and incorrect lever press response of non-RFT [non-RFT (error)] were 2.10 ± 0.38 s and 4.71 ± 1.16 s in the retention test for the simple discrimination task, 2.06 ± 0.27 s and 2.75 ± 0.62 s in the early learning stage of the FN task, and 1.71 ± 0.34 s and 3.85 ± 0.95 s in the late learning stage of the FN task, respectively. The Two-Way repeated measures ANOVA with reaction time revealed a significant trial type × stage interaction, [*F*_(2, 20)_ = 15.10, *p* < 0.001]. *Post-hoc* tests revealed that the reaction time in the RFTs was significantly shorter than that in the non-RFT (error) in the three stages (all *p* < 0.05). Moreover, the reaction time in the RFTs in the late learning stage of the FN task was significantly shorter than that in the retention test for the simple discrimination or late learning stage of the FN task (all *p* < 0.05). Further, the reaction time in the non-RFT (error) in the early learning stage of the FN task was significantly shorter than that in the retention test for the simple discrimination task and the late learning stage of the FN task (*p* < 0.05; Figure 2B).

### Hippocampal theta activity

**Figure 3:**
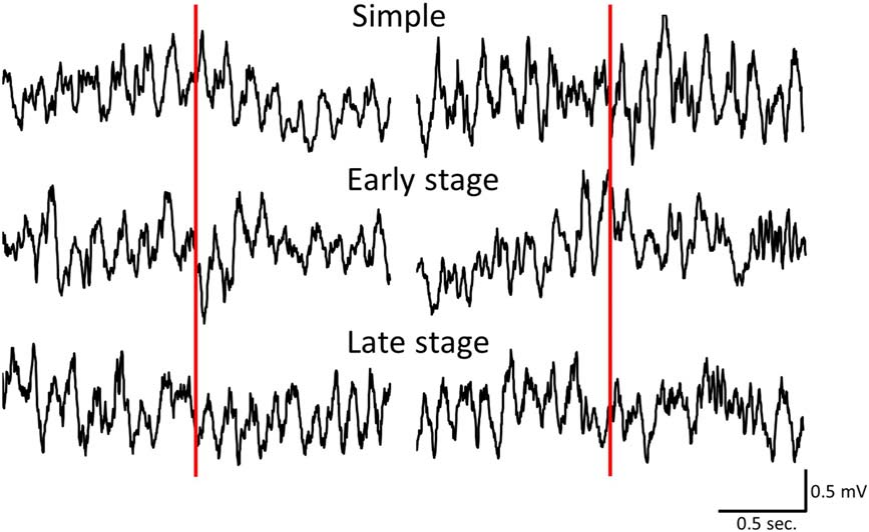
**Sample of hippocampal EEG wave during simple discrimination task (Simple), early learning stage (Early stage), and late learning stage (Late stage)**. Left panels indicated the sample of hippocampal EEG during RFT and Right panels indicated the sample of hippocampal EEG during non-RFT. Red line was at the point of stimulus presentation.

**Figure 4:**
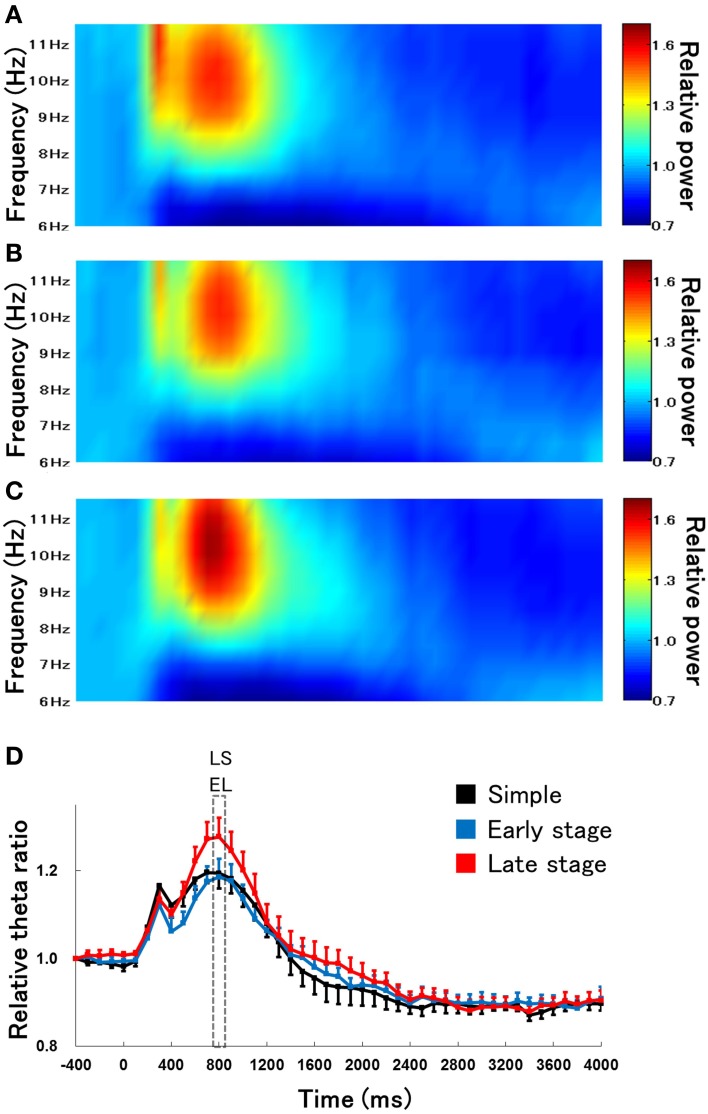
**The relative change in hippocampal theta power during RFTs during the retention test for the simple discrimination task (A), the early learning stage (B), and late learning stage (C) of the FN task**. The 0-ms period indicates the onset of stimulus presentation. The x-axis represents time (ms), and the y-axis represents frequency (Hz) in panels **(A–C)**. The period was divided into 45 sub-periods of 100 ms each. The mean hippocampal theta power from 500 to 400 ms before stimulus onset was counted as the −400-ms period (no stimuli were present and no rats pressed the lever during this period), and the relative theta power calculated for each period was normalized to that of the −400-ms period (relative theta power of each period = theta power of each period/theta power at the −400-ms period). Panel **(D)** depicts the mean hippocampal theta power between 6 and 12 Hz (Simple: simple discrimination task; Early stage: early learning stage of the FN task; Late stage: late learning stage of the FN task). The “ES” indicates a significant difference between the early learning stage of the FN task and the retention test for the simple discrimination task (ES: *p* < 0.05), the “EL” indicates a significant difference between the early learning stage and the late learning stage of the FN task (EL: *p* < 0.05), and the “LS” indicates a significant difference between the late learning stage of the FN task and the retention test for the simple discrimination task (LS: *p* < 0.05). Error bars indicate s.e.m.

We first examined the difference in the time course of changes in hippocampal theta waves during lever press response trials for RFTs between the retention test for the simple discrimination, early learning, and late learning stages of the FN task (the percentage of trials eliminated due to artifacts were 0, 3.33, and 3.00% of total RFT trials, respectively). We confirmed that typical hippocampal theta wave was represented during task (Figure 3). The Two-Way repeated measures ANOVA on relative hippocampal theta power during RFTs revealed a significant interaction between stage (retention test for the simple discrimination task, early learning and late learning stages of the FN task) × epoch [−400 to 4000 ms, each lasting 100 ms; *F*_(88, 880)_ = 2.41, *p* < 0.001] and a significant effect of epoch [*F*_(44, 440)_ = 36.20, *p* < 0.001], but no significant effect of stage [*F*_(2, 20)_ = 0.70, *n. s.*]. *Post-hoc* tests showed that there was a significant simple main effect of stage in the 700-, 800-, and 1800-ms epochs during RFTs. Multiple comparisons revealed that the hippocampal theta power increased for the 800 ms epochs during RFTs in the late learning stage of the FN task compared with the retention test for the simple discrimination task and the early learning stage of the FN task (all *p* < 0.05; Figure 4).

**Figure 5:**
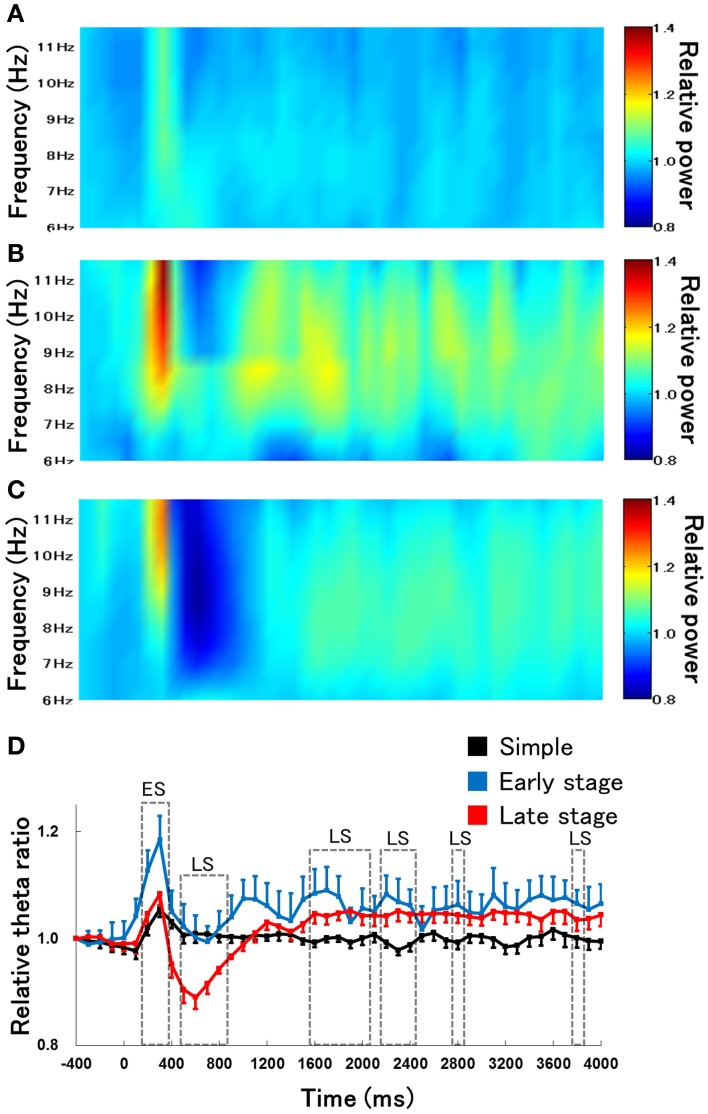
**The relative change in hippocampal theta power during non-RFTs of the retention test for the simple discrimination task (A), the early learning stage (B), and the late learning stage (C) of the FN task**. The 0-ms period indicates the onset of the stimulus presentation. The X-axis is time (ms) and the Y-axis is frequency (Hz) in panels **(A–C)**. The period was divided into 45 sub-periods of 100 ms each. The mean hippocampal theta power from 500 to 400 ms before the stimulus onset was counted as the −400-ms period (no stimuli were present and no rats pressed the lever during this period), and the relative theta power calculated for each period was normalized to that of the −400-ms period (relative theta power of each period = theta power of each period/theta power at the −400-ms period). Panel **(D)** depicts the mean hippocampal theta power between 6 and 12 Hz (Simple: simple discrimination task; Early stage: early learning stage of the FN task; Late stage: late learning stage of the FN task). The “ES” indicates a significant difference between the early learning stage of the FN task and the retention test for the simple discrimination task (ES: *p* < 0.05), “EL” indicates a significant difference between the early learning stage and the late learning stage of the FN task (EL: *p* < 0.05), and “LS” indicates a significant difference between the late learning stage of the FN task and the retention test for the simple discrimination task (LS: *p* < 0.05). Error bars indicate s.e.m.

We next examined the time course of changes in hippocampal theta activity during lever-press response inhibition trials for non-RFTs between the retention test for the simple discrimination, early learning, and late learning stages of the FN task (the percentage of trials eliminated due to artifacts were 0, 8.97, and 1.87% of total non-RFT trials, respectively). The Two-Way repeated measures ANOVA on the relative hippocampal theta power during non-RFTs showed a significant interaction of stage (retention test for the simple discrimination task, early learning, and late learning stages of the FN task) × epoch [−400 to 4000 ms, each lasting 100 ms; *F*_(88, 880)_ = 2.12, *p* < 0.001] and a significant effect of epoch [*F*_(44, 440)_ = 4.49, *p* < 0.001] but not stages [*F*_(2, 20)_ = 3.38, *p* = 0.054]. *Post-hoc* tests revealed a significant simple main effect of stage in the 200-, 300-, 500–800-, 1600–2000-, 2100–2300-, 2800-, and 3300-ms epochs during non-RFTs. Multiple comparisons revealed that hippocampal theta power increased at the 200- and 300-ms epochs during non-RFTs in the early learning stage of the FN task compared with the retention test for the simple discrimination task (all *p* < 0.05). We also found a decrease in hippocampal theta power at the 500–800-ms epochs and an increase in the theta power at the 1600–2000-, 2200–2400-, 2800-, and 3300-ms during non-RFTs in the late learning stage of the FN task compared with the retention test for the simple discrimination task (all *p* < 0.05; Figure 5).

### Hippocampal gamma activity

**Figure 6:**
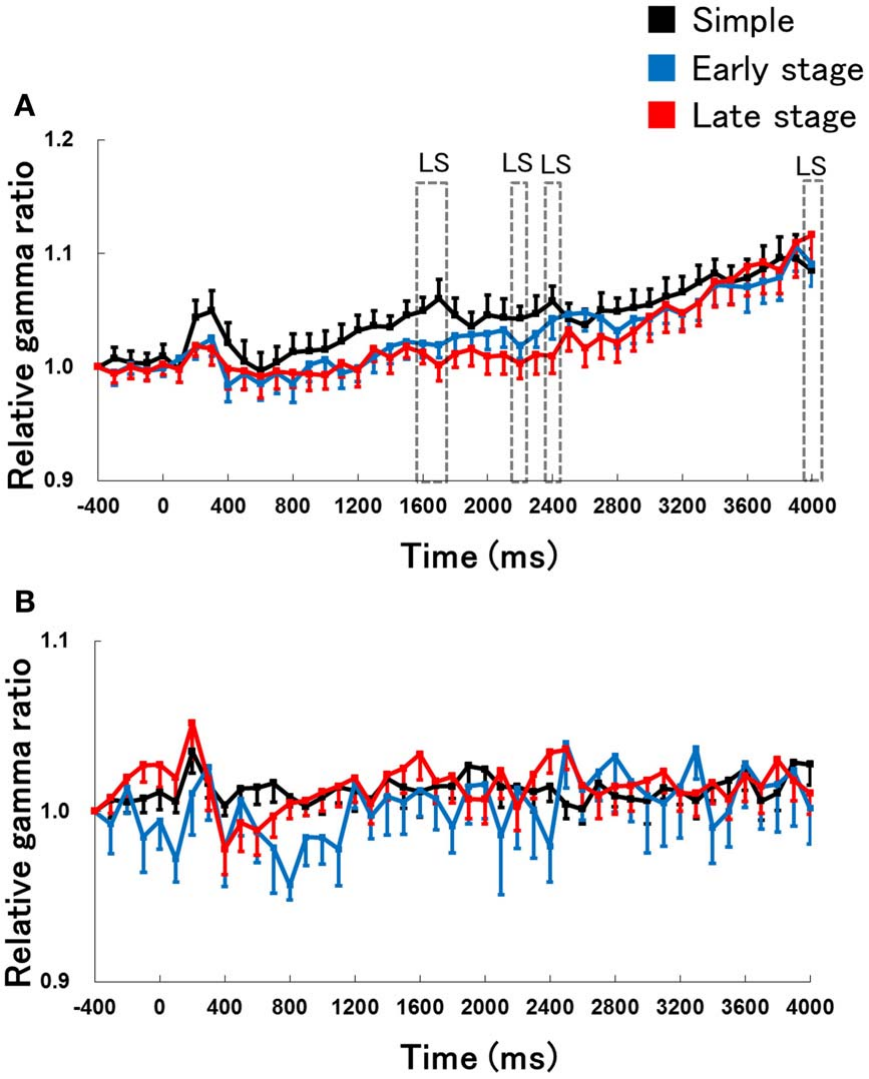
**The relative change in hippocampal gamma power during the RFTs (A) and non-RFTs (B) of the retention test for the simple discrimination task (Simple), the early learning stage (Early stage), and the late learning stage (Late stage) of the FN task**. The 0-ms period indicates the onset of stimulus presentation. The period was divided into 45 sub-periods of 100 ms each. The mean hippocampal gamma power (30–100 Hz) from 500 to 400 ms before the stimulus onset was counted as the −400-ms period (no stimuli were present and no rats pressed the lever during this period), and the relative gamma power calculated for each period was normalized to that of the −400-ms period (relative gamma power of each period = gamma power of each period/gamma power at the −400-ms period). The “LS” indicates a significant difference between the late learning stage of the FN task and the retention test for the simple discrimination task (LS: *p* < 0.05). Error bars indicate s.e.m.

We examined the difference in the time course of changes in hippocampal gamma activity (30–100 Hz) during RFTs and non-RFTs between the retention test for the simple discrimination task and late learning stage of the FN task to demonstrate that the change of hippocampal EEG activity is specific to the theta frequency band (Figure 6). The Two-Way repeated measures ANOVA on relative hippocampal gamma power during RFTs revealed a significant interaction between stage (retention test for the simple discrimination task, early learning and late learning stages of the FN task) × epoch [−400 to 4000 ms, each lasting 100 ms; *F*_(88, 880)_ = 1.84, *p* < 0.001]. *Post-hoc* tests revealed a significant simple main effect of stage in the 1600-, 1700-, 2200-, 2400- and 4000-ms epochs during RFTs. Multiple comparisons revealed that hippocampal theta gamma increased at the 1600-, 1700-, 2200-, 2400- and 4000-ms epochs during RFTs in the retention test for the simple discrimination task compared with the late learning stage of the FN task (all *p* < 0.05). The Two-Way repeated measures ANOVA on the relative hippocampal gamma power during non-RFTs showed a no significant interaction of stage (retention test for the simple discrimination task and late learning stage of the FN task) × epoch [−400 to 4000 ms, each lasting 100 ms; *F*_(88, 880)_ = 1.22, *n.s.*].

### Comparison of hippocampal theta activity between correct lever press responses for RFT and incorrect lever press responses for non-RFT on the late learning stage of the FN task

**Figure 7:**
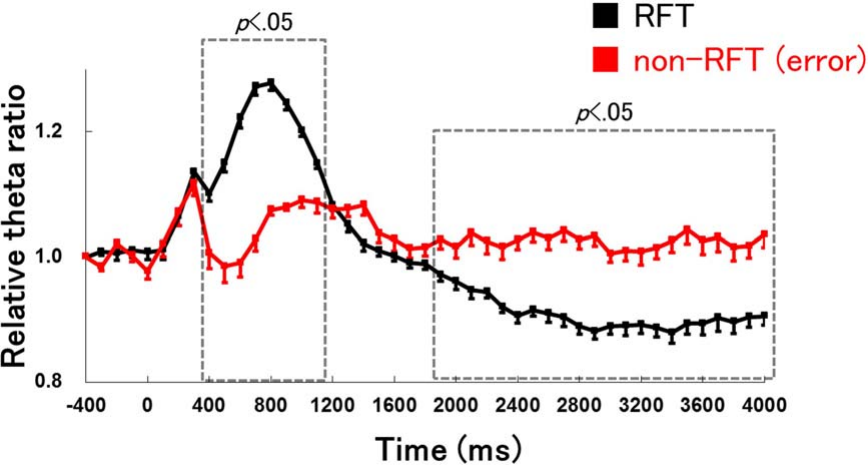
**Comparison of the hippocampal theta activity between correct lever press responses for RFTs (RFT) and incorrect lever press responses for non-RFTs [non-RFT(error)] on the late learning stage of the FN task**. The 0-ms period indicates the onset of stimulus presentation. The period was divided into 45 sub-periods of 100 ms each. The mean hippocampal theta power from 500 to 400 ms before the stimulus onset was counted as the −400-ms period (no stimuli were present and no rats pressed the lever during this period), and the relative theta power calculated for each period was normalized to that of the −400-ms period (relative theta power of each period = theta power of each period/theta power at the −400-ms period). The hippocampal theta power increased in 400- to 1100-ms epochs and decreased at 1900–4000 ms periods during the correct lever press responses for RFTs compared with during incorrect lever press responses for non-RFTs in the late learning stage of the FN task (all *p* < 0.05). Error bars indicate s.e.m.

We compared the hippocampal theta power between trials with correct lever press responses for RFT and incorrect lever press responses for non-RFTs during the late learning stage of the FN task to determine whether changes in hippocampal theta power are related to lever press movement (Figure 7). The analysis period from 400 ms before presentation of the stimulus to 4000 ms after presentation of the stimulus was divided into 45 100-ms epochs. The Two-Way repeated measures ANOVA on the relative hippocampal theta power showed a significant interaction of stage (retention test for the simple discrimination task and late learning stage of the FN task) × epoch [−400 to 4000 ms, each lasting 100 ms; *F*_(44, 484)_ = 38.82, *p* < 0.001]. Multiple comparisons revealed that hippocampal theta power increased in 400- to 1100-ms epochs and decreased in 1900–4000 ms epochs during the correct lever press responses for RFTs compared with during incorrect lever press responses for non-RFT in the late learning stage of the FN task (all *p* < 0.05).

### Comparison of hippocampal theta activity during the early learning stages of the simple discrimination and FN tasks

**Figure 8:**
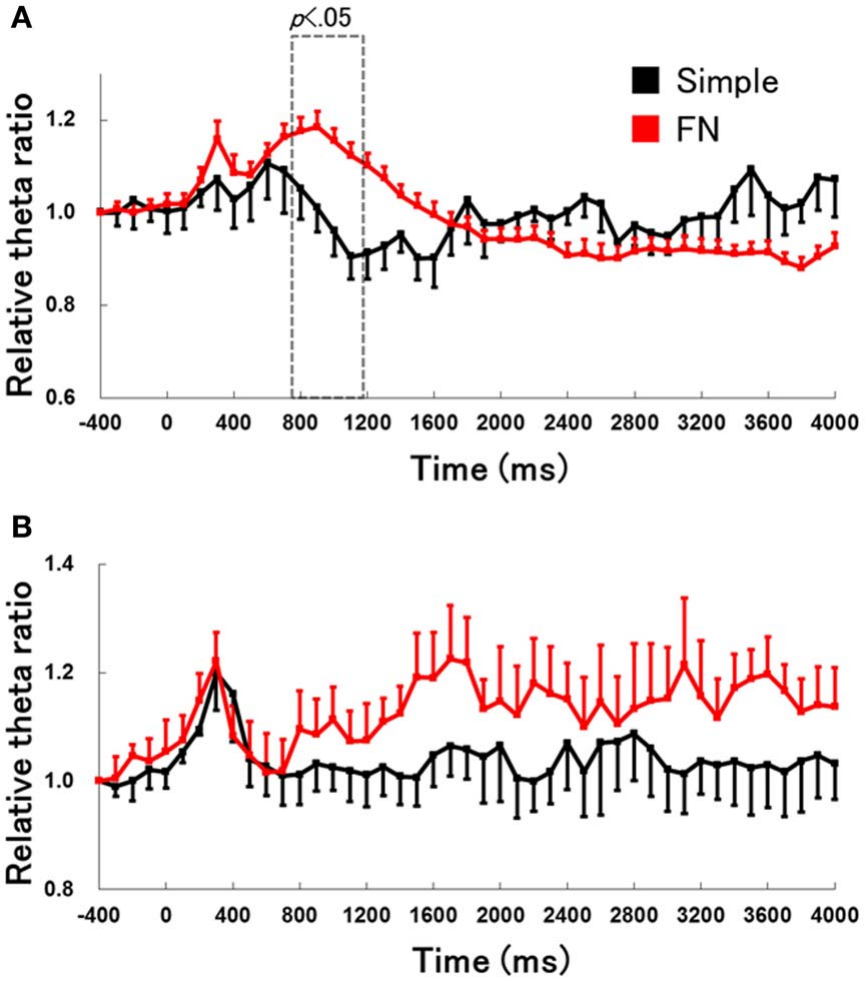
**The change in hippocampal theta power during the RFTs (A) and non-RFTs (B) of the early learning stage of the simple discrimination and FN tasks**. The 0-ms period indicates the onset of stimulus presentation. The period was divided into 45 sub-periods of 100 ms each. The mean hippocampal theta power from 500 to 400 ms before the stimulus onset was counted as the −400-ms period (no stimuli were present and no rats pressed the lever during this period), and the relative theta power calculated for each period was normalized to that of the −400-ms period (relative theta power of each period = theta power of each period/theta power at the −400-ms period). The hippocampal theta power increased in 800- to 1100-ms epochs during RFTs in the early learning stage of the FN task compared with in the early learning stage of the simple discrimination task (all *p* < 0.05). Error bars indicate s.e.m.

We compared behavioral data and hippocampal theta activity in the early learning stages between the simple discrimination and FN tasks (Figure 8). These data for the early learning stage of the simple discrimination task was used in the first three training sessions in the simple discrimination task group (*n* = 6) from our previous study (Sakimoto et al., 2013b). The Two-Way mixed ANOVA on relative hippocampal theta power during RFTs revealed a significant interaction of task (simple discrimination and FN task) × epoch [−400 to 4000 ms, each lasting 100 ms; *F*_(44, 704)_ = 4.56, *p* < 0.001]. Multiple comparisons revealed that hippocampal theta power increased at the 800–1100-ms epoch during RFTs in the early learning stage of the FN task compared with the early learning stage of the simple discrimination task (all *p* < 0.05).

A Two-Way mixed measures ANOVA on the relative hippocampal theta power during non-RFTs showed no significant interaction of task (simple discrimination and FN task) × epoch [−400 to 4000 ms, each lasting 100 ms; *F*_(44, 704)_ = 0.57, *n. s.*].

## Discussion

### Change in hippocampal theta activity and performance on the FN task after transfer from the simple discrimination task

This study showed that all rats except one required multiple sessions to learn the FN task completely, and showed impaired performance on the FN task. This impairment was demonstrated by an increase in response rates for non-RFTs, following transfer from the simple discrimination task. If the same strategy was utilized for the FN and the simple discrimination tasks, then learning on the FN task did not require multiple sessions, and task performance was retained or facilitated. Previous studies have shown that the number of sessions required for a rat to learn a task completely, differed between the FN and simple discrimination tasks (Sakimoto and Sakata, 2013; Sakimoto et al., 2013a). The results reported here support the possibility that rats use a different strategy for solving the FN and simple discrimination tasks. Furthermore, we found that hippocampal theta power transiently declined at 500- and 800-ms epochs, and then increased during response inhibition for the compound stimulus during the late learning stage of the FN task, but not during the simple discrimination task or the early learning stage of the FN task. We have previously shown that the transient decline of theta power for a compound stimulus occurred in the 500-ms epoch, with an increase in theta power for the late learning stage of the negative patterning task (Sakimoto and Sakata, 2013). Here, we revealed that hippocampal theta power declined during an incorrect compared with correct lever press response, during the FN task. We have also previously described a difference in hippocampal theta power during a negative patterning task (Sakimoto et al., 2013b). The change in hippocampal theta power found in this study is thus consistent with our previous reports (Sakimoto and Sakata, 2013; Sakimoto et al., 2013b). We therefore proposed that solving the FN task required the same hippocampal-dependent strategy as the negative patterning task.

In our previous study, we observed that hippocampal theta power did not increase for the compound stimulus part of the FN task. This is in conflict with the results reported here, which might be due to the differences in the experimental protocols used by the two studies (Sakimoto and Sakata, 2013). In the present study, training on the FN task was preceded by training for the simple discrimination task, but not by the simple discrimination task, as it was in the previous study (Sakimoto and Sakata, 2013). This study revealed that there was a difference in hippocampal theta power during RFTs between FN and the simple discrimination task, in the early stage. Moreover, the behavioral data revealed that the correct response during RFTs was retained for the simple discrimination task. We think that the difference in hippocampal theta power between these tasks reflected the degree of acquisition of the lever press response. In the non-RFT, the correct response was not retained from the simple discrimination task, and there was no difference in hippocampal theta power between the early stage of FN and the simple discrimination task. Impaired performance for non-RFTs in the FN task indicates that rats reacquire an association between response inhibition and the non-reinforced stimulus for solving the FN task. In our previous study, rats did not need to reacquire the association (Sakimoto and Sakata, 2013). Thus, we propose that reacquiring the association between response inhibition and the non-reinforced stimulus induces the increase in hippocampal theta power.

Previous studies have used lesion methodology to show that hippocampal function is not essential for solving the FN task (Solomon, 1977; Chan et al., 2003). In these studies, hippocampal lesions did not impair learning performance on this task. However, we believe that the FN task can be solved in a hippocampal-dependent and a hippocampal-independent manner. Under normal circumstances, the hippocampus is involved in solving the FN task, however, in cases where hippocampal function is impaired, or if the hippocampus is lesioned, an animal can compensate by solving this task using a hippocampal-independent strategy.

### The transient increase and decrease in hippocampal theta power during non-RFT on the FN task after transfer from the simple discrimination task

This study showed that hippocampal theta power increased during response inhibition, for compound stimuli at 200–300 ms, in the early stage of the FN task. This increase was only maintained for 200-ms periods, and this transient increase of theta power disappeared in the late stage of the FN task. Our previous study also observed a transient increase in hippocampal theta power, during the presentation of a compound stimulus in the early, 250-ms period of the negative patterning task, but not in the simple discrimination task. Moreover, this increase disappeared following acquisition of the task (Sakimoto et al., 2013b). A transient increase in hippocampal theta power has also been observed in other studies. For example, Basar-Eroglu and Demiralp (2001) showed that a transient increase in theta amplitude was induced with the expectancy of a cue that predicted the onset of a significant period, in both cats and humans. This increase of theta amplitude was also affected by the P1, N2, and P3 components of the event related potentials (ERPs). Recently, Shin (2011) observed that a transient increase occurred during presentation of a target stimulus that signaled a reward, during an auditory oddball task in rats, and this increase was coincident with the hippocampal P300 ERP. Taken together, we propose that the transient increase in hippocampal theta power reflects cognitive processing, such as attention to a stimulus that signals a reward. In addition, this study showed that hippocampal theta power decreased transiently during presentation of compound stimulus, at 500 to 800 ms, in the late stage of the FN task. Wyble et al. (2004) showed a transient decline in hippocampal theta power that reflected the cessation of approach to a reward, using a runway task in rats. In addition, Sinnamon (2005, 2006) showed a decline in hippocampal theta power, during presentation of a negative cue which suppressed approach locomotion in rats, and proposed that the decline related to the preparation or planning of behavioral inhibition. From these results, we conclude that the transient decline in hippocampal theta power is closely related to behavioral inhibition. Furthermore, some studies have shown that the interaction between theta and gamma activity plays a role in successful performance on learning and memory tasks (Tort et al., 2009; Shirvalkar et al., 2010; Li et al., 2012). We therefore examined the possibility that the transient increase or decrease in hippocampal theta power during RFTs and non-RFTs was the result of a change in power in the gamma band. During the period in which a transient increase or decrease in hippocampal theta power was observed, a change in gamma band power was not seen. However, this study showed that there was a difference of activity in the gamma band between learning stages (the retention test for the simple discrimination, the early learning, and the late learning stages of the FN task). Thus, we demonstrated the possibility that a difference in task-solving strategy between the simple discrimination and FN tasks affected the gamma band.

### Relationship between increase of hippocampal theta activity and movement during RFTs and non-RFTs

Several researchers have shown that hippocampal theta activity is strongly related to voluntary motor movements in rats, such as running, jumping, rearing, exploratory behavior, sniffing, and lever pressing (Vanderwolf, 1969; Whishaw and Vanderwolf, 1973; Wyble et al., 2004; Montgomery et al., 2009; Schmidt et al., 2013). In this study, hippocampal theta power increased at the 800-ms period during the RFT and at the 1600–2000-, 2200–2400-, 2800-, and 3300-ms during non-RFTs of the late learning stage of the FN task, as compared with the retention test for the simple discrimination task. Recently, Montgomery et al. (2009) and Schmidt et al. (2013) showed the relationship between speeds of movement and hippocampal theta power. Schmidt et al. (2013) showed that rats' hippocampal theta power was positively correlated with running speed by using plus maze task. In the current study, rats performed a lever press movement for reinforced stimulus in RFTs in order to gain the reward and we observed that well-trained rats were resting or waiting in front of the lever until presentation of stimulus. And, this study showed that the reaction time of lever press for the RFTs of the late learning stage of the FN task was the shorter than that in simple discrimination and early learning stage of the FN task. This result showed that the increase of hippocampal theta power related to speed of approach for lever and was consistent with previous studies that hippocampal theta power was affected by movement speeds. On the other hand, rats did not perform lever press movements during non-RFTs. As far as we observed, there was no difference in the rats' behavior during the any learning stages (the retention test for the simple discrimination, early learning, and late learning stages of the FN task). During the non-RFTs, most rats were resting or waiting in front of the lever. Thus, we believe that the difference in hippocampal theta activity between the simple discrimination and FN tasks was not caused by any differences in movement. However, we did not examine the relationship between hippocampal theta activity and rats' movement during learning task in details, therefore, in future study, we have need to reveal these relationship.

## Conclusion

In this study, we examined whether solving the FN and simple discrimination tasks use the same strategy. The result showed that performance of the FN task was impaired with transfer from the simple discrimination task and that hippocampal theta activity differed between the FN and simple discrimination tasks. Thus, we argue that solving the simple discrimination and FN tasks use different strategies.

## Author contributions

Yuya Sakimoto met all for four criteria for authorship recommended from ICMJE. He, specifically, made an important contribution to the conception and design of the work, and helped construct the discussion in this work.

### Conflict of interest statement

The authors declare that the research was conducted in the absence of any commercial or financial relationships that could be construed as a potential conflict of interest.
